# Condensate dynamics at the synapse: Phase separation tunes presynaptic function

**DOI:** 10.1371/journal.pbio.3003201

**Published:** 2025-06-11

**Authors:** Janine Lützkendorf, Stephan J. Sigrist

**Affiliations:** 1 Freie University Berlin, Institute for Biology and Genetics, Berlin, Germany; 2 Charité Universitätsmedizin, NeuroCure Cluster of Excellence, Charitéplatz, Berlin, Germany

## Abstract

Liprin-α and RIM form liquid phase-separated condensates that organize presynaptic architecture and fine-tune neurotransmitter release. This Primer explores a new study in PLOS Biology analysing how these dynamic assemblies shape synaptic fidelity and what their disruption might mean in neurodevelopmental disease.

## Teaser

A new study in PLOS Biology shows that Liprin-α and RIM form condensates which not only scaffold presynaptic protein architectures but also fine-tune vesicle priming and release dynamics.

Efficient neurotransmission depends on the proper assembly and organization of presynaptic active zones (AZs), composed of a conserved set of scaffolding and regulatory proteins. These include Liprin-α, RIM, RIM-BP, ELKS, Munc13, and voltage-gated calcium channels, which coordinate the docking, priming, and fusion of synaptic vesicles in response to calcium influx [1,2]. While the molecular roles of many AZ proteins have been well established, the mechanisms that dynamically regulate their spatial organization and interaction have remained elusive. Several recent studies have proposed that liquid–liquid phase separation (LLPS) may underlie the assembly of AZ components into dynamic, membrane-less condensates, particularly involving ELKS, RIM, RIM-BP, and Munc13 ([3–6]. In this issue of PLOS Biology, Jin and colleagues [7] advance this model by providing the first structural and functional dissection of the Liprin-α/RIM interaction—a core scaffolding module—and linking it to phase separation and neurotransmission.

Jin and colleagues dissect the molecular interaction between Liprin-α2 and RIM1, identifying the minimal region in Liprin-α2 (CC2N, the N-terminal half of coiled-coil domain 2) that binds to the C2B domain of RIM1. Using analytical size-exclusion chromatography and isothermal titration calorimetry, they show that deletion of CC2N abolishes RIM1 binding, while deletion of the C-terminal region (CC2C) disrupts ELKS interaction, indicating that distinct domains mediate specific partner binding. High-resolution X-ray crystallography revealed the structure of the Liprin-α2-CC2N/RIM1-C2B complex. The interaction is stabilized by a β-sheet interface involving both polar and hydrophobic contacts, including salt bridges and hydrogen bonds. Mutation of conserved residues in RIM1 (e.g., R1201Q, mimicking RIM4) disrupts the interaction, highlighting its specificity. A secondary contact interface also supports oligomeric complex formation, albeit with a lesser impact on affinity.

Liprin-α2 and RIM1 form large multivalent complexes capable of LLPS. Jin and colleagues observed that the RIM1-C2B domain forms dimers, promoting higher-order assemblies with Liprin-α2. In vitro LLPS assays showed that the presence of Liprin-α2 (CC1 + CC2) substantially increased RIM1 condensate formation. Disrupting the Liprin-α2/RIM interface via point mutations (E334R or R346E) reduced droplet size and number, directly linking binding interface integrity to phase behavior. These findings suggest that LLPS of the Liprin-α/RIM complex may serve as a scaffold for concentrating AZ components such as ELKS, Munc13, and calcium channels, supporting efficient vesicle priming and release.

To probe the functional consequences of disrupting this interaction, the authors re-expressed wild-type or mutant Liprin-α2 constructs in human neurons lacking all Liprin-α isoforms. Surprisingly, both wild-type and interface-disrupting mutants rescued synaptic puncta formation, suggesting that the Liprin-α2/RIM1 interaction is not essential for structural assembly of AZs. However, electrophysiological recordings told a different story. Liprin-α knockout neurons showed abolished spontaneous synaptic release, which was restored only by wild-type—but not mutant—Liprin-α2. These data indicate that while the Liprin-α/RIM interface may be dispensable for assembly, it is critical for functional neurotransmitter release. Further analyses revealed that this interaction also regulates the size of the readily releasable pool of synaptic vesicles. Thus, LLPS-driven Liprin-α/RIM condensates not only scaffold presynaptic architecture but also fine-tune vesicle priming and release dynamics.

The Liprin-α/RIM complex additionally modulates voltage-gated calcium channels localization by influencing ELKS1 and RIM1 condensate behavior. Disruption of their interaction increased ELKS1 accumulation within RIM1 droplets and reduced voltage-gated calcium channels recruitment, suggesting that Liprin-α/RIM complexes spatially coordinate calcium channel positioning to ensure tight coupling between action potentials and vesicle fusion. Using optogenetic tools (e.g., channelrhodopsin-assisted mapping), Jin and colleagues demonstrate that this coordination ensures precise release timing—a hallmark of synaptic fidelity.

Importantly, this interaction has implications beyond basic neuroscience. Jin and colleagues tested several disease-associated mutations in the CC2N domain of Liprin-α2—including E328K, L348F, and A350S—which have been identified in patients with autism spectrum disorders, intellectual disability, and epilepsy [8,9]. Mutations like E328K and L348F strongly impaired RIM binding, while A350S had a more modest effect. These mutations likely disrupt condensate formation and vesicle release, offering a mechanistic explanation for their association with synaptic dysfunction and neurodevelopmental disorders.

**Fig 1:**
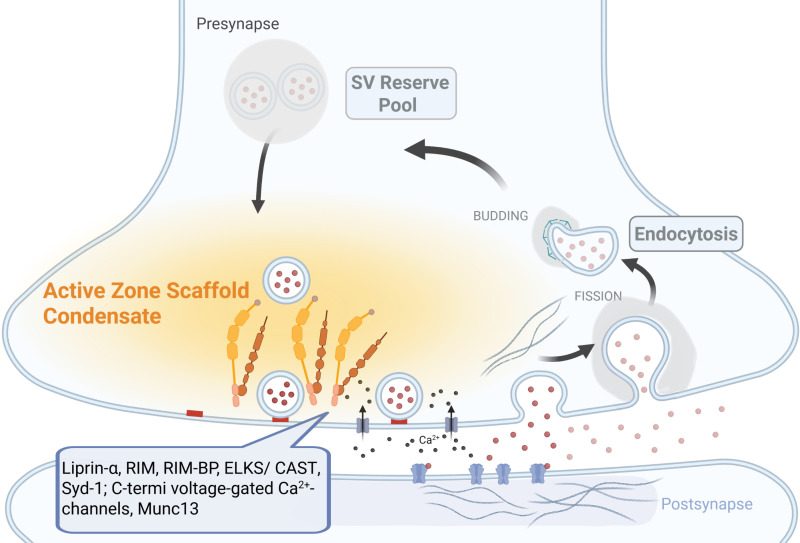
Intrinsically disordered proteins and phase-separated condensates coordinate presynaptic active zone structure and function. The presynaptic terminal organizes neurotransmission through dynamic and spatially structured protein assemblies. Synaptic vesicles (SVs) are distributed between a docked pool at the AZ and a reserve pool. Precise coupling between docked SVs and voltage-gated calcium channels enables rapid release, a process supported by an underlying AZ scaffold. Jin and colleagues propose that scaffold components, including Liprin-α and RIM, through their multifaceted intermolecular interaction, assemble into condensates via liquid-liquid phase separation (LLPS) that organize the molecular and functional architecture of the AZ. These dynamic assemblies help cluster SV release machinery while remaining responsive to synaptic activity. Thus, LLPS-driven Liprin-α/RIM condensates not only scaffold presynaptic architecture but also fine-tune vesicle priming and release dynamics. Following exocytosis, SV membranes are retrieved at the periactive zone through clathrin-mediated endocytosis, coordinated by a second network of IDR-containing proteins. These proteins can similarly form endocytic condensates that drive membrane curvature, sorting, and fission. Together, AZ and endocytic condensates represent two molecularly distinct but spatially coordinated compartments that sustain high-throughput synaptic activity while maintaining structural plasticity. Liprin-α and RIM form condensates which not only scaffold presynaptic protein architectures but also fine-tune vesicle priming and release dynamics. Understanding the principles of condensate dynamics opens new avenues to explore how synaptic transmission, plasticity, and structural AZ stability are getting co-regulated—and how their dysregulation may contribute to neurological disease. Created with BioRender.com.

By resolving the Liprin-α/RIM interface at atomic detail and functionally dissecting its contribution to synaptic release, Jin and colleagues build a compelling case for LLPS as a modular mechanism to organize and tune the functional output of presynaptic terminals. In contrast to prior studies that linked phase separation primarily to AZ formation, this work reveals a decoupling between AZ structure and function, highlighting how condensate composition and interface integrity shape neurotransmitter release. These findings position LLPS as a key organizing principle at the presynaptic AZ, critical for synaptic fidelity and adaptable function (Fig 1).

Yet several questions remain: How is condensate formation regulated by neuronal activity or signaling cues? How do LLPS-based mechanisms integrate with parallel scaffolding pathways to ensure structural and functional resilience? And, could dysregulation of synaptic condensates contribute to disease beyond the mutations identified here? While LLPS has been well characterized in vitro, genetic strategies to finely modulate phase behavior in vivo remain challenging. Notably, in a recent study [10], using in vivo single-molecule imaging approaches have begun to bridge this gap by visualizing phase-transition-like dynamics at remodeling As, such as nanocluster compaction of ELKS and Cacophony channels during synaptic potentiation.

This work further suggests that condensates formed by RIM and Liprin-α may play a dual role—serving as molecular scaffolds while also promoting functional release. Vesicle exocytosis from RIM/Liprin-α condensates is less sensitive to slow calcium buffering (EGTA), consistent with nanodomain coupling to voltage-gated calcium channels. By contrast, condensates lacking proper phase-separating interactions show increased EGTA sensitivity, pointing to impaired spatial coupling. These findings imply that RIM/Liprin-α condensates help tune both the positioning and efficiency of release machinery, offering a self-organizing mechanism that ensures temporal precision in synaptic transmission. This and similar mechanisms may also underlie the striking diversity of presynaptic release properties across neuronal types, suggesting that condensate material properties—such as viscosity, molecular exchange rates, or fusion competency—could be modulated to tune synaptic output to specific circuit demands. The study by Jin and colleagues makes a valuable contribution by elucidating the structural basis of the Liprin-α2/RIM1 interaction and demonstrating its potential role in organizing presynaptic components. Their use of stem cell-derived neurons and in vitro reconstitution provides important mechanistic insight. However, the work does not address whether these molecular interactions have functional consequences in vivo. The reliance on simplified cellular systems and high protein concentrations to elicit phase separation raises questions about the physiological relevance of the findings under native synaptic conditions. Moreover, the absence of in vivo electrophysiological or behavioral analyses limits the ability to assess how these mechanisms contribute to synaptic transmission or circuit function in an intact nervous system.

A particularly interesting aspect of the study is the identification of disease-associated Liprin-α2 mutations (L348F, E328K) that disrupt RIM1 binding and are linked to neurodevelopmental disorders. These findings suggest a direct molecular mechanism by which synaptic transmission is impaired. The structural insights may guide strategies to restore or mimic the Liprin-α2/RIM1 interaction, or to modulate their LLPS behavior, with potential therapeutic relevance for conditions such as autism or epilepsy.

Understanding the principles of condensate dynamics opens new avenues to explore how synaptic transmission, plasticity, and structural AZ stability are getting co-regulated—and how their dysregulation may contribute to neurological disease.

## Abbreviations

AZ: active zone
LLPS: liquid–liquid phase separation
SVs: synaptic vesicles
